# Lyme borreliosis and medical wandering: what do patients think about multidisciplinary management? A qualitative study in the context of scientific and social controversy

**DOI:** 10.1186/s12879-024-09194-3

**Published:** 2024-03-22

**Authors:** Alice Raffetin, Costanza Puppo, Amal Chahour, Assia Belkasmi, Elisabeth Baux, Solène Patrat-Delon, Pauline Caraux-Paz, Julie Rivière, Sébastien Gallien

**Affiliations:** 1 Department of Infectious Diseases, Tick-Borne Diseases Reference Center of Paris and the Northern Region, Centre Hospitalier Intercommunal de Villeneuve-Saint-Georges, 40 Allée de La Source, 94190 Villeneuve-Saint-Georges, France; 2 EpiMAI Research Unity, Laboratory of Animal Health, Anses-National Veterinary School of Alfort, 7 Av. du Général de Gaulle, 94700 Maisons-Alfort, France; 3 DYNAMIC Research Unity, UPEC-Anses, 8 Rue du Général Sarrail, 94000 Créteil, France; 4 Department of Psychology, University Lyon II, France, UMR 1296, 86 Rue Pasteur, 69007 Lyon, France; 5 Department of Public Health, University of Versailles Saint-Quentin en Yvelines, 55 Avenue de Paris, 78000 Versailles, France; 6 Department of Infectious Diseases, Tick-Borne Diseases Reference Center of the Eastern Region, Brabois Hospital, University Hospital of Nancy, Rue du Morvan, 54500 Vandœuvre-Lès-Nancy, France; 7 Department of Infectious Diseases, Tick-Borne Diseases Reference Center of the Western Region, University Hospital of Rennes, 2 Rue Henri Le Guilloux, 35033 Rennes Cedex 9, France; 8 Department of Infectious Diseases, Tropical Medicine and Immunology, University Hospital Henri Mondor, 1 Rue Gustave Eiffel, 94000 Créteil, France

**Keywords:** Lyme borreliosis, Satisfaction, Multidisciplinarity, Shared information

## Abstract

**Introduction:**

To answer to patients’ medical wandering, often due to “unexplained symptoms” of “unexplained diseases” and to misinformation, multidisciplinary care centers for suspected Lyme borreliosis (LB), such as the 5 Tick-Borne Diseases (TBDs) Reference Centers (TBD-RC), were created a few years ago in France, the Netherlands and Denmark. Our study consisted of a comprehensive analysis of the satisfaction of the patients managed at a TBD-RC for suspected LB in the context of scientific and social controversy.

**Methods:**

We included all adults who were admitted to one of the TBD-RC from 2017 to 2020. A telephone satisfaction survey was conducted 12 months after their first consultation. It consisted of 5 domains, including 2 free-text items: “What points did you enjoy?” and “What would you like us to change or to improve?”. In the current study, the 2 free-items were analyzed with a qualitative method called reflexive thematic analysis within a semantic and latent approach.

**Results:**

The answer rate was 61.3% (349/569) and 97 distinctive codes from the 2-free-text items were identified and classified into five themes: (1) multidisciplinarity makes it possible to set up quality time dedicated to patients; (2) multidisciplinarity enables seamless carepaths despite the public hospital crisis compounded by the COVID-19 pandemic; (3) multidisciplinarity is defined as trust in the team’s competences; (4) an ambivalent opinion and uncertainty are barriers to acceptance of the diagnosis, reflecting the strong influence of the controversy around LB; and (5) a lack of adapted communication about TBDs, their management, and ongoing research is present.

**Conclusion:**

The multidisciplinary management for suspected LB seemed an answer to medical wandering for the majority of patients and helped avoid misinformation, enabling better patient-centered shared information and satisfaction, despite the context of controversy.

**Supplementary Information:**

The online version contains supplementary material available at 10.1186/s12879-024-09194-3.

## Introduction

Many patients suspected of having Lyme borreliosis (LB) may experience diagnosis wandering and difficult carepaths [1–5]. Indeed, the diagnosis and management of LB — the most frequent tick-borne disease (TBD) in Europe and the United States, caused by the bacteria *Borrelia burgdorferi* sensu lato [6, 7] — may be challenging for several reasons, such as the painless nature of the tick bite (making it difficult to detect and recall), the wide range of clinical pictures of LB (sometimes resembling other pathologies) [8], the possible presence of disabling subjective symptoms at all stages of the disease [9–11], a microbiological diagnosis relying mainly on an indirect serological test [12], and complex management, based not only on antibiotic therapy for 14 to 28 days but also on the symptomatic management of various symptoms [1–5, 11, 13, 14]. In some patients, nonspecific symptoms may persist despite appropriate antibiotic therapy for LB, referred to as post-treatment Lyme disease syndrome (PTLDS), and these patients may wonder if *Borrelia* is still active, if post-infectious symptoms are present, or if a different diagnosis is responsible for these symptoms. This situation may result in misunderstandings for some patients who are not convinced that an antibiotic therapy for 14–28 days, based on scientific evidence, is enough to eradicate *Borrelia*. This doubt may be reinforced by the observation that post-infectious diseases and their management are not so well documented and that differential diagnoses, despite extensive diagnostic procedures and treatments, may not result in the resolution of symptoms (e.g., chronic inflammatory or neurodegenerative diseases). Here, we can clearly identify how the “long-term antimicrobial” concept emerged and has become one of the major points of the controversy today.

To improve the health-care organization of LB, a French national care plan for LB was launched in 2016 that favored the creation of the five Tick-Borne Diseases Reference Centers (TBD-RC) to manage patients presenting with LB via a multidisciplinary approach (joint endeavor of infectious diseases physicians, rheumatologists, neurologists, psychologists, etc.). The need for these structures was raised by patients in a previous study exploring their perceptions, representations, and experiences of their disease and care-paths [15]. They expressed a wish for change, especially with better listening, greater recognition of symptoms, and simpler care-paths.

Other countries, such as the Netherlands and Denmark, have initiated similar care organizations since 2010 [1–3], showing a European awareness of the need to optimize the management of complex LB and its differential diagnoses. These multidisciplinary experiences revealed a low prevalence of confirmed LB (10%–20%) and the multiplicity of differential diagnoses [1–5]. Nonetheless, we previously showed that the majority of patients (80.7%), independent of their final diagnoses (classified as confirmed LB, possible LB, PTLDS, or another diagnosis), had favorable clinical outcomes 12 months after their first consultation at the TBD-RC of Paris and the Northern Region [4]. Most of the patients (84.8%) were satisfied with this multidisciplinary health-care organization, and 80.2% accepted their diagnoses [16]. The high satisfaction with the information issued by the doctors was the foundation of the satisfaction with the management, highlighting the importance of the doctor–patient relationship and of the shared medical decision that may help reduce health misinformation. However, these preliminary quantitative findings about the opinions of the patients concerning these multidisciplinary structures for LB have not yet been studied with a psychosocial and qualitative approach, preventing conclusions on the underlying reasons for their satisfaction and dissatisfaction*.* Studies under the social sciences are necessary to improve comprehension of the patients’ expectations, apparent paradoxes, and points of satisfaction and dissatisfaction. They would also help better understand the sources of misinformation that may have led to medical wandering or to dissatisfaction with the TBD-RC. This is why this study adopted a psychosocial theoretical approach to provide a comprehensive analysis of the satisfaction and dissatisfaction of the patients managed at the TBD-RC for suspected LB in the context of scientific and social controversy.

## Methods

### Population, setting and intervention

This study used data from a prospective cohort study of all patients consulting at the TBD-RC of Paris and the Northern Region for suspected LB from December 1, 2017 to December 1, 2020. As previously published [4], a medical file was requested prior to consultation, enabling the team to analyze all previous consultations/hospitalizations, tests, and treatments. There were no limitative criteria to accept patients. After a multidisciplinary one-hour consultation, a medical summary was given to the patients with the first orientation of the care pathway. If needed, additional tests were performed and complementary specialized medical advice sought. Patients with diagnoses associated with LB were classified as having confirmed LB (exposure to tick bite – not necessarily a proven tick bite –, evocative clinical signs, and positive serology), possible LB (exposure to tick bite, evocative clinical signs, and negative serology or atypical signs and positive serology), or PTLDS/sequelae (persistent symptoms after a previous confirmed LB already treated as recommended) [8, 17]. The other patients were considered as having “other diagnoses,” made by a doctor specialized in the field. An orientation in the adapted medical department was offered to every patient. The patients were regularly reassessed through a medical consultation at 3, 6, and 12 months. A telephone-based satisfaction survey was conducted independently from the staff consulting at the TBD-RC 12 months after the first consultation at the center.

### Satisfaction survey

The satisfaction survey covered five domains: (1) reception; (2) care and quality of management; (3) information and explanations given to the patients; (4) current medical condition after the management at the TBD-RC compared to the previous one and acceptance of the final diagnosis; and (5) overall appreciation (Additional File 1). There were 15 items, including 13 rated items (between 0 and 10) and 2 free-text items: “What points did you enjoy?” and “What would you like us to change or to improve?” This questionnaire was inspired by the MedRisk instrument and adapted to our setting [18, 19]. The patients’ associations with LB and other diseases (such as HIV) were consulted to confirm that it met their expectations. The quantitative analyses of the 13 rated items were previously published [16]. Satisfaction with the management was defined as a score ≥ 7 and dissatisfaction as a score < 7. Acceptance of the diagnosis was defined as a “yes” answer and the absence of acceptance was defined as a “partially” or a “no” answer. In the current study, only the 2 free-text items were analyzed.

### Statistical analyses

The socio-demographic characteristics of satisfied and dissatisfied patients were compared. The categorical variables were reported as proportions and percentages and the continuous variables as medians with interquartile ranges (IQRs). The categorical variables were compared using chi-squared or Fisher’s exact test, as appropriate. The continuous variables were compared between the groups via the t-test or Mann–Whitney test, as appropriate.

### Thematic analysis

To analyze the two free-text items, the qualitative method of reflexive thematic analysis (TA) was used [20, 21]. The aim of TA is to develop “themes” across a dataset that address a research question. First, a semantic approach was chosen to report the explicit content of the data. Second, a latent approach was applied to enable to understand the participants’ points of view with a comprehensive approach and to highlight concrete applications in a health-care relationship [20–22]. We previously listed our mind-dependent truths to ensure a critical analysis of the data, including our own subjectivity. In this study, patterns were generated by the main investigator (AR) through a rigorous process. The TA started after the full dataset had been collected as this study is part of a preexisting cohort.

First, data familiarization was achieved by reading the answers of the patients several times and by creating a word cloud using Word Art (version 4.7.7). It enabled us to present the occurrence of the words, proportional to their size in the figure, to visually highlight the positive and negative aspects associated with the TBD-RC by the participants. Only common names, adverbs and adjectives were kept.

Second, systematic data coding, with a semantic approach, was performed—going line by line through each transcript. These codes were refined as the analyses advanced, with a latent approach as well, to report concepts and hypotheses underpinning the explicit content of the data. Inductive notes were taken by AR and mirrored to her clinical experience, as she is an infectious diseases physician. Another investigator (AB) also independently coded the transcripts. This enabled better reflexivity, especially as AB had no clinical experience as she was a public health worker. The two perspectives were compared to bring out their similarities and differences and it led to a consensus.

Third, theme development was used to classify the codes into initial themes, with the attempt to avoid being influenced by the results of preexisting studies so as to adhere to an inductive approach, all while still recognizing inevitable subjectivity [20]. Thematic maps were used to develop thinking and to visualize specific themes, as the analyses progressed. The previous inductive notes helped at this stage, and frequent returns to the raw data were necessary to keep a faithful interpretation and to develop a coherent qualitative analysis.

Fourth, the themes were reexamined several times as an iterative process, and the results were written all along the TA, which helped improve the definitions and naming the themes.

### Approval of the ethics committee

The local ethics committee of the University Hospital of Créteil, France, approved this research (N°2021–02-03). All the included patients gave informed consent to the use of their medical data for research purposes prior to their management at the TBD-RC of Paris and the Northern Region and to the satisfaction questionnaire. The research sponsor signed a commitment to comply with “Reference Methodology MR004” of the French Data Protection Authority (CNIL, 2,216,096 v 0, December 10, 2019).

## Results

Of the 569 patients admitted to the TBD-RC of Paris and the Northern Region, 349 (61.3%) answered the satisfaction questionnaire 12 months after their management. Among the 220 non-responders, 10 refused (lack of time), 7 had medical reasons, and 203 were not reached by phone after 3 calls.

### Characteristics of the patients

We compared the characteristics of the patients who were satisfied or dissatisfied with the management at the TBD-RC and of the patients who accepted or denied their final diagnoses (Table 1). Overall, 297 (85.1%) patients were satisfied with their management (score ≥ 7) and 52 were dissatisfied (score < 7). Moreover, 280 (80.2%) accepted their final diagnosis, 29 (8.3%) partially accepted it, and 40 (11.5%) did not accept it. There were more patients with confirmed LB and with shorter durations of symptoms in the groups “satisfied patients” and “diagnostic acceptance” than in the groups “dissatisfied patients” and “no diagnostic acceptance” (respectively, *p* = 0.016 and *p* = 0.007 and then *p* = 0.021 and *p* = 0.017). Patients who did not accept their diagnoses had received non-recommended antibiotics more often than patients who accepted their diagnoses (*p* = 0.036).

**Table 1:** Comparison of the characteristics of the satisfied and dissatisfied patients with their management at the TBD-RC and of the patients who accepted or not their diagnosis

Characteristics of the patients	Total *N* = 349 (%)	Satisfied patients *N* = 297 (%)	Dissatisfied patients *N* = 52 (%)	*p*-value	Diagnostic acceptance *N* = 280 (%)	No Diagnostic acceptance *N *= 69 (%)	*p*-value
**Age, (years), median [IQR]**	48 [35,62]	49 [35, 62]	42.5 [33,58]	0.393	48 [34,62]	48 [37,60]	0.558
**Male**	146 (41.8)	122 (41.1)	24 (46.2)	0.494	117 (41.8)	29 (42.0)	0.971
**Lifestyle**				0.676			0.708
Home in a rural area	72 (20.6)	62 (20.9)	10 (19.2)		56 (20.0)	16 (23.2)	
Employment in rural areas/forest	17 (4.9)	14 (4.7)	3 (5.8)		15 (5.4)	2 (2.9)	
Forest-based leisure activities	249 (71.4)	213 (71.7)	36 (69.2)		201 (71.8)	48 (69.6)	
No exposure	11 (3.2)	8 (2.7)	3 (5.8)		8 (2.9)	3 (4.4)	
**Past history of tick bite**	234 (67.1)	201 (67.7)	33 (63.5)	0.551	189 (67.5)	45 (65.2)	0.718
**Past history of erythema migrans**	97 (27.9)	90 (30.3)	7 (13.7)	**0.015**	82 (29.3)	15 (22.1)	0.233
**Patients referred by a physician with a letter**	313 (89.7)	264 (88.9)	49 (94.2)	0.218	250 (89.3)	63 (91.3)	**0.015**
General Practitioner	241 (69.1)	200 (67.3)	41 (78.9)		183 (65.4)	58 (84.1)	
Specialist physician	59 (16.9)	51 (17.2)	8 (15.4)		55 (19.6)	4 (5.8)	
Emergency unit physician	13 (3.7)	13 (4.4)	0 (0.0)		12 (4.3)	1 (1.5)	
No letter, patient self-referral	36 (10.3)	33 (11.1)	3 (5.8)		30 (10.7)	6 (8.7)	
**Duration (days) of chief complaints prior to consultation at TBD-RC, median [IQ 25,75]**	425.5 [140.5, 1208.5]	416 [133, 1155]	804 [230, 1679]	**0.021**	382 [128, 1174]	561 [249, 1437]	**0.017**
Positive serological test*	111 (31.8)	98 (33.0)	13 (25.0)	0.118	91 (32.5)	20 (28.9)	0.869
**Final diagnosis at TBD-RC**				**0.016**			**0.007**
Confirmed LB	48	47 (15.8)	1 (1.9)		47 (16.8)	1 (1.5)	
Possible LB	31	29 (9.8)	2 (3.9)		24 (8.6)	7 (10.1)	
PTLDS/sequelae	34	27 (9.1)	7 (13.5)		24 (8.6)	10 (14.5)	
Other diagnoses	236	194 (65.3)	42 (80.8)		185 (66.1)	51 (74.0)	
**Antibiotic therapy prescribed before TBD-RC**	228 (65.3)	192 (64.7)	36 (69.2)	0.522	179 (63.9)	49 (71.0)	0.268
Antibiotic therapy > 4 weeks	71 (20.3)	55 (18.5)	16 (30.8)	**0.043**	49 (17.5)	22 (31.9)	**0.008**
Non-recommended treatments **	61 (17.5)	47 (15.8)	14 (26.9)	0.052	43 (15.4)	18 (26.1)	**0.036**

*LB* Lyme borreliosis, *PTLDS *Post-Treatment Lyme Disease Syndrome, *IQR* Inter quartile range, *TBD-RC*  Tick-Borne Diseases Reference Center, *A positive serological test = *IgM and/or IgG positive in ELISA and WB if the tick bite occurred less than 6 weeks ago; or IgG positive only if the tick bite occurred more than 6 weeks ago*, *ELISA* Enzyme-Linked Immunosorbent Assay, *WB* Western-Blot; **Non-recommended treatment: > 8 weeks of antibiotics and/or associated antimicrobials.

### Word cloud: Data familiarization

**Fig. 1:**
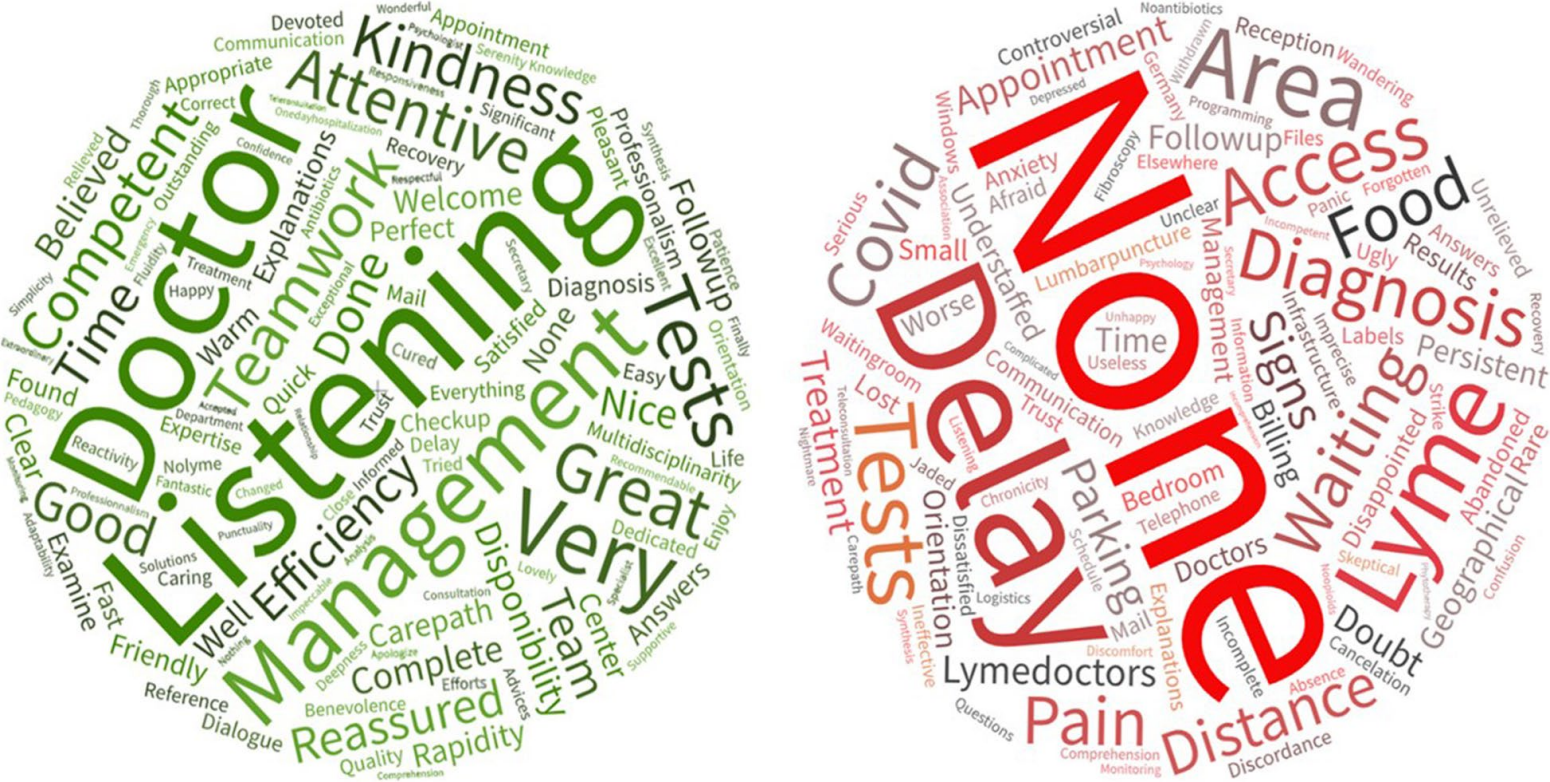
Word cloud describing positive and negative aspects of the TBD-RC of Paris and the Northern Region from patients’ perspective

The occurrence of the words used to answer the two free-text items are presented in Fig. 1.

To the question “What points did you enjoy?”, 134 different words were cited by the patients with high occurrences (> 40 repetitions of the same word), for example “listening” (*n* = 79), “management” (*n *= 54), “attentive” (*n* = 43) etc. The word “none” was cited seven times. To the question “/What would you like us to change or to improve?”, 101 different words were cited by the patients, but the most cited was “none” (*n* = 110). The other words had lower occurrences than in the positive aspects, such as “delay” (*n* = 23), “not Lyme” (*n* = 16), etc.

### Systematic data coding and Identified themes

**Fig. 2:**
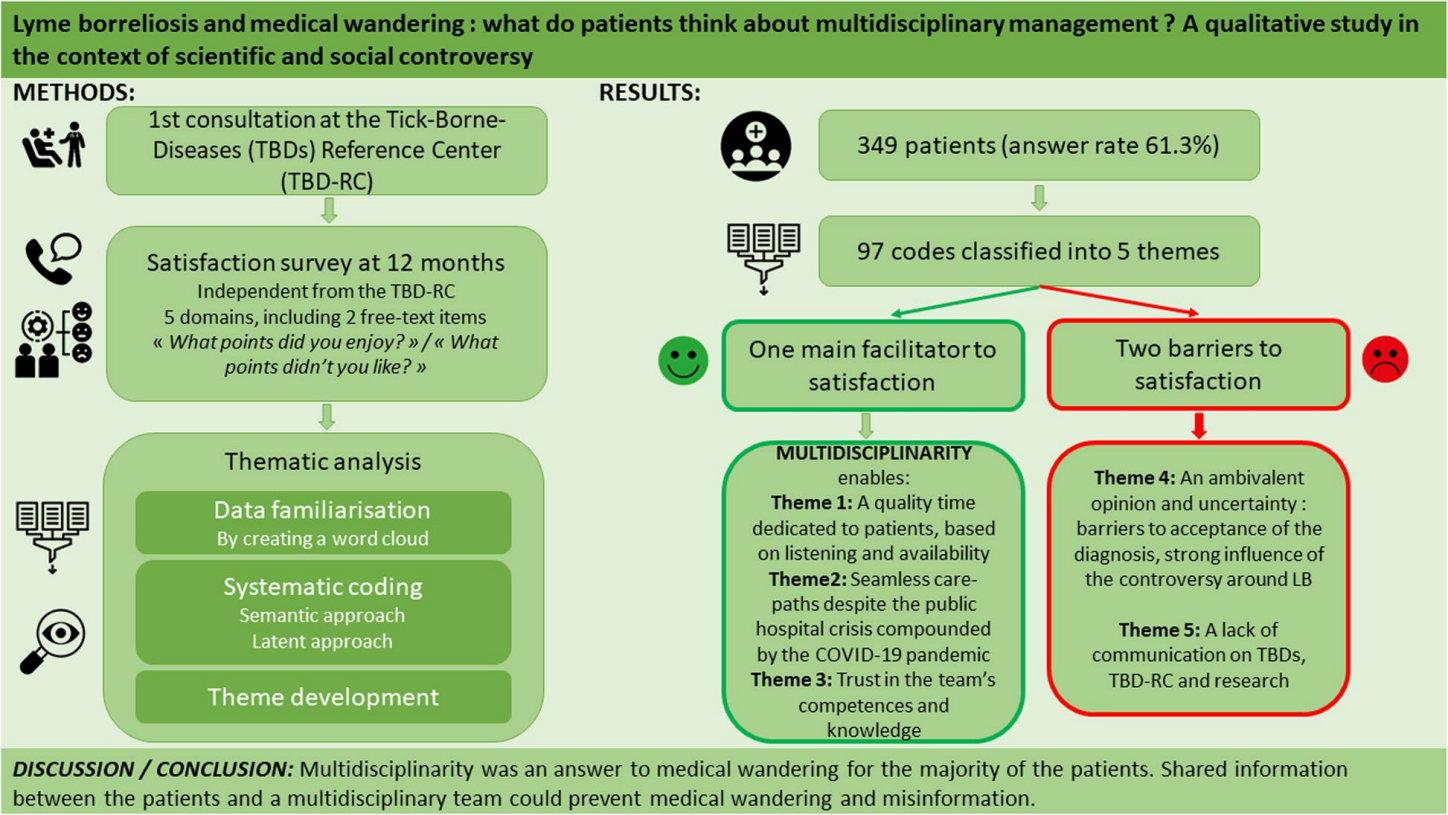
Summary of the methods and main findings about the reasons for satisfaction and dissatisfaction of the patients managed at the Tick-Borne Diseases Reference Center of Paris and the Northern Region

Ninety-seven codes were identified: 41 in the analyses of the positive aspects and 55 in the negative aspects. The main facilitator to patient satisfaction was multidisciplinarity, leading to three themes of satisfaction (themes 1–3), and the two identified barriers were the controversy (theme 4) and the lack of adapted communication (theme 5) (Fig. 2).

The codes were classified in five main themes:

- *1. Multidisciplinarity makes it possible to set up quality time dedicated to patients.* First, privileged time allowed better listening and more availability from the staff and encouraged benevolence: “The physician took the time to listen to me and to understand my personal history.” Second, this dedicated time improved the quality of the patient–doctor relationship based on shared medical decisions and shared information: “The doctor believed me. He took me seriously.” and “I had a good contact. I was able to ask my questions, and I had clear answers.” Multidisciplinarity enabled coming up with different solutions to the same health problem, which broadened the medical options for the patients: “I received great advice adapted to my case and positive comments.” However, a few patients found that they had received too much information. The medical synthesis given at the end of the day had helped them read the information again and understand it at their own pace. On the contrary, 10 patients “went back home with their questions” because they were overwhelmed by the number of experts they had seen in one day. They didn't dare to ask their questions, and therefore the answers did not appear in their synthesis. Finally, this dedicated time appeared to have a positive impact on the patients’ quality of life: “It changed my life all throughout my care-paths at the TBD-RC and even now” and “I felt supported and did not suffer anymore from medical wandering.” The participants had a good opinion about this survey, which demonstrated the doctors’ “concern for patients’ physical and mental well-being.”
- *2. Multidisciplinarity enables seamless care-paths despite the public hospital crisis compounded by the COVID-19 pandemic*. The majority of the patients were satisfied with the short delay for the first appointment, but a few experienced difficulty in initial appointment setting. The management at the TBD-RC was reported as “fast, efficient, and safe”: “Very reactive team” and “I didn’t know we could get this far in a single day.” A prolonged follow-up and close monitoring reassured the patients who felt supported. It enabled to confirm the health state improvement or to revise the diagnosis and management if necessary: “It was important to have regular follow-up, even by teleconsultation, especially during the pandemic.” The patients appreciated having their medical synthesis with the diagnosis and the offered care-paths, given in person by the doctor, at the end of the day. It also helped their general practitioner (GP). Despite being admitted to an old hospital with the premises under construction, modified patient circuits inside the hospital caused by COVID-19, and a geographical setting in suburban Paris, this multidisciplinary structure helped the patients reach difficult-access medical specialties: “I live in a medical desert. So at last, I could have all I needed in one place, even if it was far from home.”
- *3. Multidisciplinarity enables trust in the team’s competences and knowledge.* Multidisciplinarity favored skill and knowledge sharing, enabling expertise for complex cases in terms of diagnosis and management. Long discussions between the professionals and the patients about the latter’s past medical history and their symptoms, such as fatigue or pain, as well as meticulous physical examinations by different specialists reassured the patients and gave them confidence in the team: “The doctors were really competent, with a very good knowledge of LB but also of so many other diseases! They shared their hypothesis, and they finally found my diagnosis.” Moreover, most of the patients reported that the maximum means had been implemented for them—“all the medical investigations had been performed”—which helped them accept the final diagnosis, even in the absence of LB, ending their medical wandering. However, four patients refused to consult the psychologist, considering this unnecessary, whereas most of the patients appreciated it: “For the first time, someone was finally telling me I was not crazy, it was not in my head, and I was not depressed” and “I think a psychological support should be systematically offered. No one emerge unscathed from medical wandering.” Finally, the respect of guidelines and protocols was perceived as reassuring: “To have a doctor who was compliant with the guidelines was essential for me. I was afraid of all the crazy things I had read on the internet.”
- 4. *An ambivalent opinion and uncertainty are barriers to acceptance of the diagnosis, reflecting the strong influence of the controversy around LB*. First, some patients experienced persistent symptoms despite “satisfactory” management. Some of them felt lost between opposing narratives: “They found another disease, and I was well treated. However, another physician in town said I still had Lyme and prescribed me an additional six months of antibiotics. I don't know who I’m supposed to trust” and “I feel fully healed. But I feel trapped by the controversy, you see. Am I really cured?” Second, some patients felt destabilized by a negative serological test at our center, often ruling out the diagnosis of LB: “I got a positive test in Germany and not in France. The doctor explained to me that we had the same tests in both countries and that the one I had done was not recommended. Who should I trust?" Third, some patients questioned the final diagnosis offered by the TBD-RC in the absence of LB: “Good listening, availability, investigations done—but no Lyme. So I went to a Lyme doctor who confirmed LB.” Fifteen patients challenged the physician’s diagnosis, interrupted their care-paths, and consulted a Lyme doctor (physician offering alternative care-paths, including “long-term antimicrobial” treatment). Seven reported that the physician at the TBD-RC did not believe in “chronic Lyme disease”. It was attributed to a “lack of knowledge about LB” from doctors who were “bound by the High Authority of Health and not free to prescribe non-recommended treatments.” Finally, controversy was sometimes perceived as harmful to patients and as a barrier to progress in terms of both care-paths and research: “The information is too controversial. We don’t need that when we’re not well. We just want to trust and get better. Formal care-paths and no more talking about it!” Two patients felt so lost by the opposing views that it affected their trust in the health-care system and worsened their medical wandering: “I'm so confused about all these speeches—‘on again, off again.’ All the doctors I’ve seen here and there, all the treatments I’ve taken over the last year for nothing... I would rather suffer alone, but I no longer trust physicians.”
- *5. A lack of adapted communication about TBDs, their management in reference centers, and ongoing research projects is present.* “Not enough communication about these structures and TBDs” and “People have anxiety out of ignorance. Physicians and patients alike! LB is not well recognized. They should be trying to communicate massively on this and their reference centers”. Some patients reported that they had mainly found fake information on LB and TBDs beforehand to learn about these structures, leading to difficult care-paths, described as “a lack of time” and “a lack of money.” This lack of clear communication about recommended carepaths resulted in patient anxiety or doubt about whom they should see and trust. Of the 71 patients who had experienced alternative carepaths before the TBD-RC, 70% were satisfied with our structure. Some patients felt that LB was “not attractive to the majority of physicians,” and therefore, “the research [was] not developed” to address the lack of knowledge, especially on persistent symptoms and diagnostic tests.

## Discussion

### Summary of the main findings

**Fig. 3:**
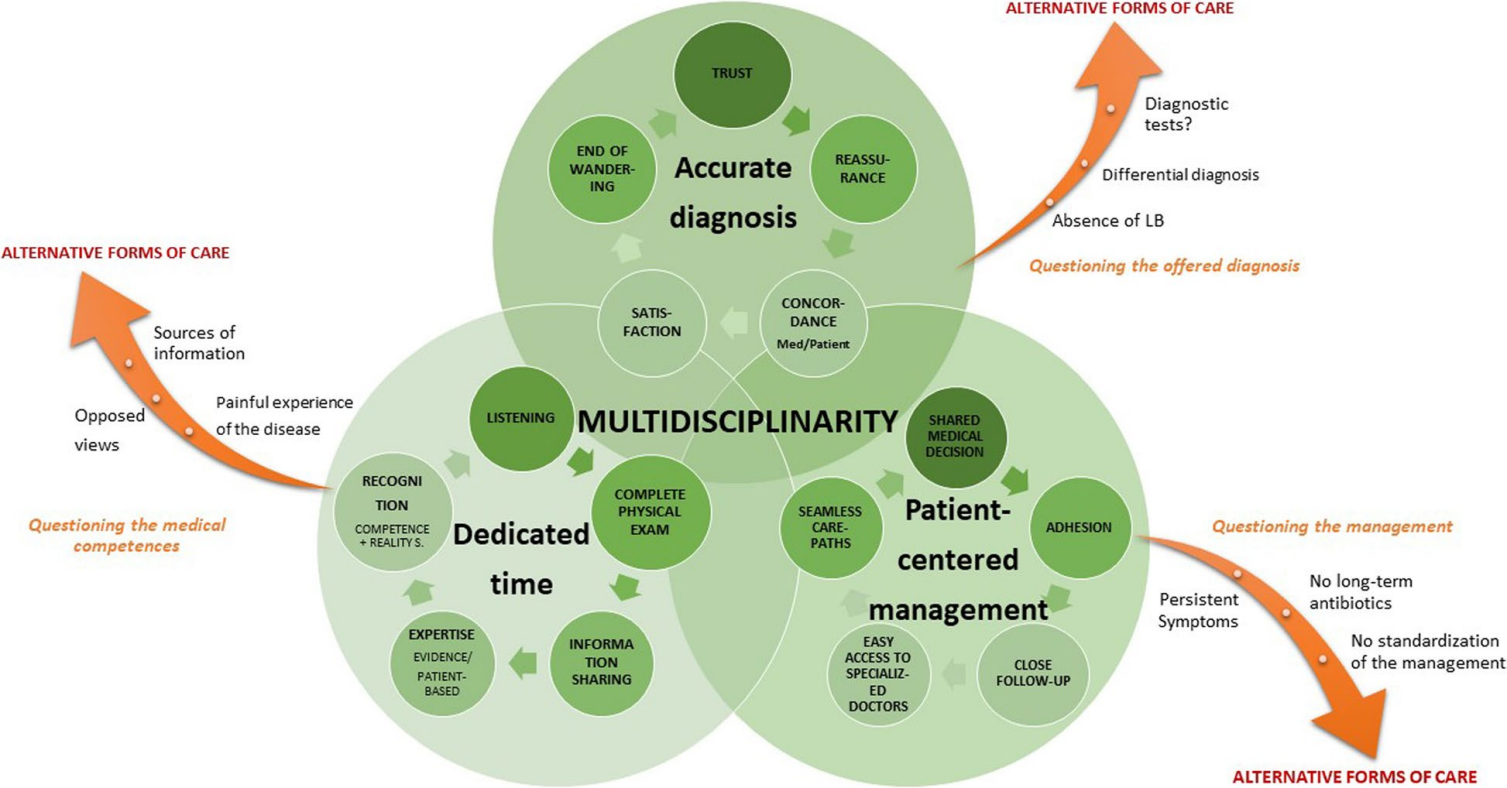
Modeling of the satisfaction with the diagnosis and management of patients with a suspected Lyme borreliosis in a multidisciplinary center such as the TBC-RC of Paris and the Northern region

Our study qualitatively analyzed the satisfaction of patients about their diagnosis and management of suspected LB in a multidisciplinary center such as the TBD-RC of Paris and the Northern region. We showed that multidisciplinarity enabled quality dedicated time to patients (with thorough listening, shared information, and evidence-based and patient-based expertise), an accurate diagnosis (meaning the end of medical wandering), and patient-centered management based on shared medical decisions and seamless care-paths, as modeled in Fig. 3. However, the underlying controversy on LB seemed to provoke ambivalence and uncertainty in some patients, especially in case of the absence of LB or of the presence of persistent or unexplained symptoms, causing the patients to question the medical competences, the offered diagnosis, and the management (Fig. 3).

### Meaning of the study

#### An answer to medical wandering for the majority of the patients

Previous studies about the suspicion of LB in social sciences had already reported a need for better listening, better recognition, and easier pathways that could be offered by multidisciplinary structures and attentive practitioners [15, 23]. This study demonstrated that we can meet those reported expectations with the TBD-RCs. Moreover, these multidisciplinary structures may help patients reach difficult-access medical specialties in a one-day hospitalization period. Thanks to the two previous works about this same cohort [4, 16] and this one, we tried to identify parameters to define medical wandering before the TBD-RC and during the management at the TBC-RC (Table 2). In the future, it might be of importance to measure the end of medical wandering after the TBC-RC with objective parameters.

**Table 2:** Parameters to define medical wandering before the TBD-RC and during the management at the TBC-RC

Parameters of medical wandering before the TBD-RC	Parameters of medical wandering during the TBD-RC
• Duration of symptom > 1.4 years before the TBD-RC	• Diagnostic delay > 15 days within the TBD-RC
	• Moderate satisfaction with care and medical management at the TBD-RC
• Multiplicity of suspected diagnoses at the beginning of their management	
	• Lack of acceptance of the diagnosis offered by the TBD-RC
• History of antibiotic therapy > 2 months or combination of anti-infective agents	
	• Moderate satisfaction with the carepaths within the TBD-RC
• Alternative carepaths before the TBD-RC	• Moderate satisfaction with the information exchanged and explanations given by the medical team
• Disinformation prior to the TBD-RC (access to unverified sources of information on TBD)	
• Lack of knowledge about TBD by some doctors, potentially experienced by patients as a misunderstanding of their symptoms	• Patients feeling lost between conflicting medical discussions about tests, treatments, etc
	• Non-specific symptoms in the foreground
• Non-specific symptoms in the foreground	

#### Dissatisfaction in a few patients, often associated with the controversy

Our results showed that some participants experienced uncertainty about their health condition, which may give rise to many questions and social representations [24] to understand the causes and consequences of their disease [25]. The process of attributing symptoms to an explanation influences the patients’ health behaviors [25–27] and changes over time given the influence of media and the patients’ loved ones [28]. Blaxter [29] introduced the idea of an “illness without disease” to focus on the eventual gap between the subjective experience of symptoms and objective biomedical categorizations. Many social science studies dealt with individuals living with medically unexplained symptoms and psychosocial factors [30–33]: feelings of stigma and delegitimization, medical nomadism, and significant impacts on one’s social, physical, and mental quality of life.

Moreover, some patients who were already aware of the controversy before their admission at the TBD-RC were skeptical about the existence of potential differential diagnoses, about the diagnostic tests in France, and about the recommended treatments. They had often experienced medical wandering in the conventional health-care system for years. As shown in other studies, they were “hyper-informed” and felt competent [32]. Multidisciplinarity was essential to allow dedicated time both quantitatively (with at least one hour of consultation) and qualitatively (shared discussions with several specialists). It enabled listening to them and understanding their medical histories, previous care-paths, sources of information, and beliefs. Most of the patients who had arrived “dissatisfied” at the TBD-RC were finally satisfied with the diagnosis and management and appreciated the long knowledge-sharing discussions (both lay and scientific knowledge). Thirty-five out of 61 were still disappointed, including 15 who accessed Lyme doctors. Nonetheless, as in other studies, some patients experienced alternative care-paths with no satisfactory answer prior to the TBD-RC (e.g., “too much treatments for nothing,” “the cost was too high”) [15, 33]. Those who turned to us for a second opinion mostly accepted their diagnoses (70.5%) [16]. Indeed, as already shown, the patients first sought an improvement in their health and not just a diagnostic name [15]. Diagnosis is not only a form of categorization but also a process to produce knowledge [34–36]. Individuals are not passive receptors of biomedical labels, but they can also shape them [34] depending on the different social groups involved (e.g., health professionals, other patients, their families) [35].

#### Shared information between the patients and a multidisciplinary team could prevent medical wandering and misinformation

As previously demonstrated, a well-delivered information by physicians was a key element of management satisfaction, confirming the importance of shared medical decisions to meet the patients’ expectations and reduce misinformation [16]. Concordant opinions between the doctor and the patient resulted in higher satisfaction and better care outcomes [16]. This qualitative approach thus confirms these previous findings.

Since medical information sharing needs to be considered with a social and contextual perspective [36] and not only with a cognitive one, the controversy around LB should be taken into account. According to a previous survey, about 33% of GPs had experienced difficulty with “hyper-informed” patients with a suspicion of LB for two main reasons: their own lack of deep knowledge and their uncertainty about the quality of the patient’s information sources [32]. An Indian survey conducted among 92 GPs revealed four types of “hyper-informed” patients: 5% were “completely informed patients” (having verified and accurate information, well applied to their symptoms), 81% were “misguided” (having verified and accurate information, not correctly applied to their symptoms) or “misinformed patients” (not having authentic information, not applied correctly to their symptoms), and 7% were “confused patients” who were not correctly informed (unauthentic information applied to irrelevant symptoms) [37]. Through their care-paths, patients can move from one category to another, changing from a state of overconfidence to disillusionment. The physician’s role is not to convince them but to allow them to make an informed decision with genuine information tailored to their symptoms. Open dialogue, source verification, understanding of the flow of information over the internet, consideration of the power and counter-power issues in the physician–patient relationship, and data analysis are key elements to making informed shared medical decisions.

Other studies highlighted that the uncertainty about LB from health-care providers was perceived by patients as an incomprehension of their symptoms (“questions with no answers”), even a lack of recognition (“symptoms in my head”), and was often responsible for the feeling of abandonment and for negative perceptions of the conventional health-care system [15, 23, 38, 39]. Multidisciplinarity, enabling precise and patient-centered answers, may have helped change the patients’ perceptions in a positive way in this study and to empower the patients to make genuinely shared decisions.

### Implication for practice and research: a need for concrete answers

#### Better communication with the general population could prevent medical wandering and misinformation about TBD

Forest-Bérard et al*.* suggested a novel approach of “trained ambassadors” to raise tick and LB risk awareness among outdoor visitors and workers in Canada [40]. This approach especially helped reach the at-risk population. A recent French study showed good knowledge of the population about preventions against tick bites and about LB [41]. However, Slunge et al*.* showed discrepancies between knowledge and the risk perception about LB [42]. Dernat and Johany [43] highlighted the need for a socio-spatial approach to study tick bite risk perceptions. New coordinated initiatives with higher impact are needed to better inform the general population such as described in Table 3.

**Table 3:** Summary of reported negative points and concrete solutions suggested

Reported Problems	Proposed Solutions
**A need for patient-centered information**	
*Two patients found they had received too much information*	Medical synthesis systematically given at the end of the day to ensure patients can read the information again and understand it at home
*Ten patients found they had not received enough information and went back home with their question*	Creation of a bulleted list to help patients to prepare for their appointments at TBD-RC, including one specifying “*I prepare and write down my questions before the consultation*”
*Two patients did not dare ask their questions as they were overwhelmed by the number of experts they had seen in one day*	Patients information sheet about TBD-RC sent prior to their arrival
**A need to facilitate appointments**	
*Fifteen patients complained about a lack of punctuality from doctors*	Patients informed that their appointment will occur within a time range rather than at a specific time (e.g. between 9:30 am and 11:30 am)
*Twenty-six patients described a difficult initial appointment setting*	- Notification on the website of the hospital: “*All the appointments are given by email after having received the medical documents of the patients. There is no possibility to take an appointment by phone for new patients”* - Involvement of general practitioners to refer their patients, using tele-expertise
**Questioning proposed care paths**	
*Four patients refused to consult the psychologist considering it was unnecessary*	Implementation of the information sheet to explain why we offer a systematic consultation with a psychologist, sharing the experience of satisfied patients
*Seven patients felt lost between opposing narratives*	-Discussion groups between patients and health professionals set up on controversial issues to help patients access verified sources to form their own opinion - Psychological support systematically offered
**A need for a better communication**	
*Six patients reported a lack of communication about TBDs and difficulties to assess the reliability of the information sources*	-A national day on LB was held for the first time in France in May 2023. The target audience is the general population - The TBD-RCs website was launched in March 2022 to provide information on TBDs and the reference centers to the general population and physicians, relying on mediatisation (82,942 views recorded as of January 10, 2024). This may help direct patients to consensual care pathways - National guidelines about recommended care-paths in France were published in 2022, under the aegis of the High Authority of Health, resulting from a consensus between the patient associations and official scientific societies. The target groups are physicians and the general population. This may help direct patients to consensual care pathways
*Twenty-five patients reported a lack of communication about TBD-RCs and official care-paths*	
*Three patients reported a lack of research about TBDs*	Systematic communication of the results of research projects to patients: -Flyers/Posters in the waiting room, “*What’s up to date about TBDs? What’s up to come?*” -Newsletter about the French national cohort -Discussion groups with patients

#### Concrete answers at an individual level

Concrete solutions for the main negative aspects reported by patients were listed Table 3.

#### Elaboration and assessment of a generalizable satisfaction survey about the management of TBD

The results of this study were presented both to the patients’ representatives of the TBD-RC of Paris and the Northern Region and to the teams of the other TBD-RCs. It implemented the discussion and allowed to improve this satisfaction survey (Table 4.) for the future and to generalize its use in all the TBD-RCs in France to assess its relevance.

**Table 4.**
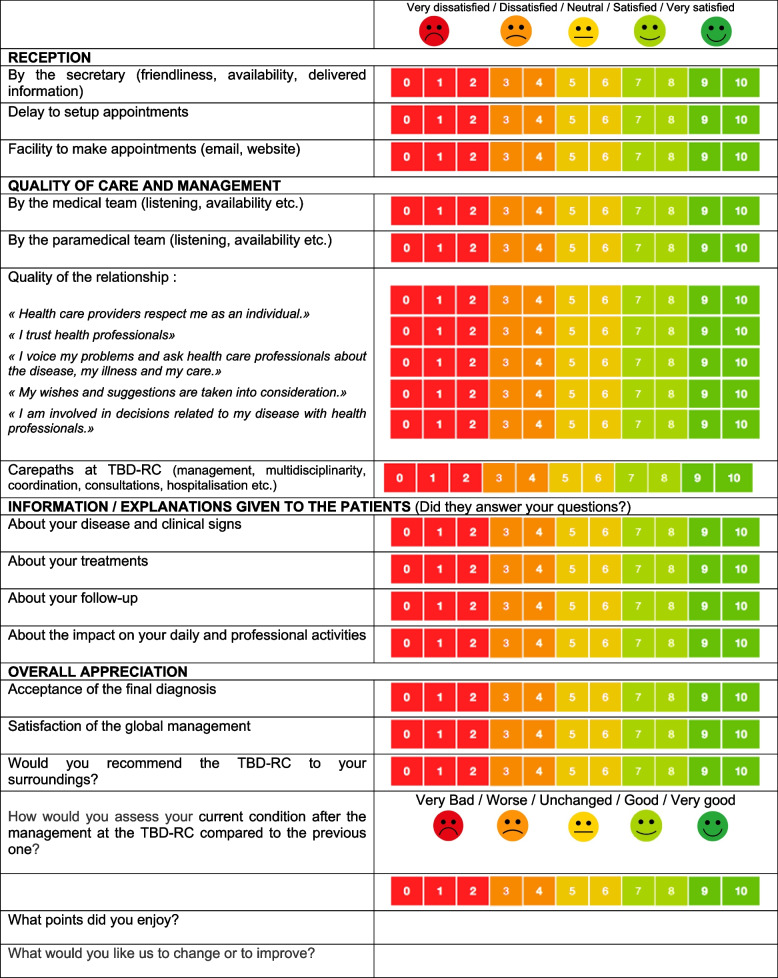
New proposal for a satisfaction survey about diagnosis and management for suspected Lyme borreliosis in a multidisciplinary structure

### Strengths and limitations of the study

The main strength of this study is that it used mixed methods (statistical and thematic analysis), enabling a deep and holistic analysis of the diagnosis and management satisfaction of the patients experiencing these multidisciplinary structures. The large number of patients empowered our findings. The telephone survey was carried out by a clinical research technician (AC) who is not involved in the patients’ care, which guaranteed freedom of speech. The double and blinded analysis of the data by two different researchers with different skills enabled better reflexivity and a critical analysis of the data, including our own subjectivity.

The main limitation of this study is its monocentric design, so the results reflect the opinion of the patients of one TBD-RC only. To balance this point, results were compared with those of other pathologies, and other TBD-RCs were asked to improve this survey so as to generalize its use in the future. We may have overestimated the patients’ satisfaction as the satisfied patients may have been more likely to answer our questions. Nonetheless, in a sensitivity analysis, we found no difference between the characteristics of the patients who answered and those who did not [16]. Moreover, medical wandering being a subjective notion, we considered in this work that a patient who accepted the diagnosis and who was satisfied with the management was not anymore in medical wandering. It would have been interesting to directly ask the question to every patient to assess the validity of this hypothesis. In addition, the telephone survey implied note-taking reflecting as much as possible the reality of the discourse of the patients, and we tried as much as possible to keep faithful to the exact words used by the patients or we tried to avoid reducing their opinions. Finally, the data was collected in French. Translation required much more than the most exact rendition of individual words as well as their meaning and context.

## Conclusion

This qualitative study assessing the satisfaction of patients with suspected LB demonstrated that a multidisciplinary structure such as the TBD-RC seemed to resolve medical wandering for the majority of the patients and helped avoid misinformation, which are both major ethical issues. Remaining uncertainty, influenced by the context of scientific and social controversy, was the main barrier to the patients’ satisfaction. Multidisciplinary centers may enable better communication that should be developed at all levels: toward individuals (patient-centered shared information), toward the general population (information on TBDs), and toward physicians (information on TBDs and TBD-RCs). Other studies are warranted to evaluate more deeply patient satisfaction in other multidisciplinary centers for LB in France and in Europe.

### Supplementary Information

**Supplementary Material 1. **

## Data Availability

The datasets used and/or analyzed during the current study are available from the corresponding author on reasonable request.
