# Supplementary material for: Lyme borreliosis and medical wandering: what do patients think about multidisciplinary management? A qualitative study in the context of scientific and social controversy
Source: BMC Infect Dis. 2024 Mar 22;24:344. doi: 10.1186/s12879-024-09194-3 (PMC10958838; doi:10.1186/s12879-024-09194-3)
Supplement: Supplementary file 1 — Supplementary Material 1. [file 12879_2024_9194_MOESM1_ESM.docx]

**SATISFACTION SURVEY**

**Tick-Borne Diseases Reference Center of Paris and the Northern Region**

|  | Very dissatisfied / Dissatisfied / Neutral / Satisfied / Very satisfied  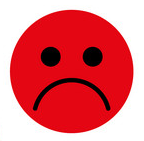 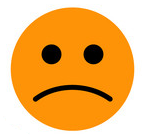 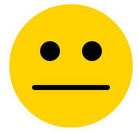 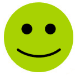 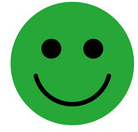 |
| --- | --- |
| **RECEPTION** | |
| By the secretary | 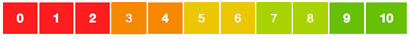 |
| **QUALITY OF CARE AND MANAGEMENT** | |
| By the medical team | 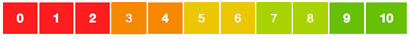 |
| By the paramedical team | 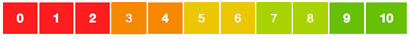 |
| Responsiveness and compassion to  patients | 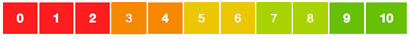 |
| Care path at TBD-RC | 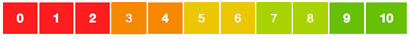 |
| **INFORMATION / EXPLANATIONS GIVEN TO THE PATIENTS** (have they answered your questions?) | |
| Given by the secretary | 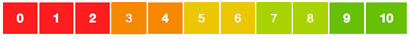 |
| Given by the paramedical team | 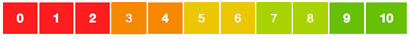 |
| Given by the medical team | 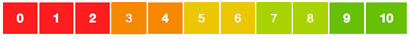 |
| **OVERALL APPRECIATION** | |
| Satisfaction of the final diagnosis | 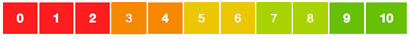 |
| Satisfaction of the global  management | 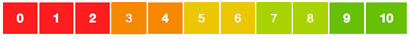 |
| Would you recommend the TBD-RC  to your surroundings? | 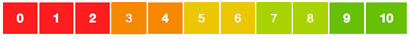 |
| What points did you enjoy? |  |
| What would you like us to change or  to improve ? |  |
| **CURRENT MEDICAL CONDITION** | |
| Acceptance of the final diagnosis | □ Yes □ Partially □ No |
| How would you assess your current  condition after the management at the  TBD-RC compared to the previous  one? | Very Bad / Worse / Unchanged / Good / Very good  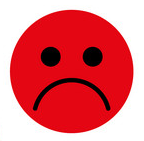 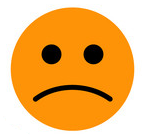 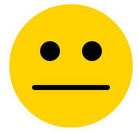 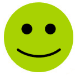 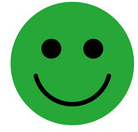  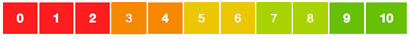 |
